# Study of the effect of nano-sized precipitates on the mechanical properties of boron-added low-carbon steels by neutron scattering techniques

**DOI:** 10.1107/S0021889808020943

**Published:** 2008-08-16

**Authors:** B. S. Seong, Y. R. Cho, E. J. Shin, S. I. Kim, S.-H. Choi, H. R. Kim, Y. J. Kim

**Affiliations:** a HANARO Center, Korea Atomic Energy Research Institute, PO Box 105, Yuseong, Daejeon 305-600, Republic of Korea; b POSCO, 699 Kumho-dong, Kwangyang-Shi, Cheonnam 545-090, Republic of Korea; c Department of Materials Science and Metallurgical Engineering, Sunchon National University, 540-742, Republic of Korea

**Keywords:** neutron diffraction, small-angle neutron scattering (SANS), boron-added low-carbon steel, elongation, cementite, precipitates, particle tracking autoradiography

## Abstract

Small-angle neutron scattering (SANS) and neutron powder diffraction (ND) techniques were used to study quantitatively the effect of nano-sized precipitates and boron addition on the mechanical properties of low-carbon steels. SANS was used to evaluate nano-sized precipitates, smaller than about 600 Å in diameter, and ND was used to determine the weight fraction of the cementite precipitates. Fine core–shell structured spherical precipitates with an average radius of ~50 Å, such as MnS and/or CuS, surrounded by BN layers were observed in the boron-added (BA) low-carbon steels; fine spherical precipitates with an average radius of ~48 Å were mainly observed in the boron-free (BF) low-carbon steels. In the BA steels, the number of boron precipitates, such as BN, Fe_3_(C,B) and MnS, surrounded by BN layers increased drastically at higher hot-rolling temperatures. The volume fraction of the fine precipitates of the BA steels was higher than that of the BF steels; this difference is related to the rapid growth of the BN layers on the MnS and CuS precipitates. Boron addition to low-carbon steels resulted in a reduction in strength and an improvement in elongation; this behaviour is related to the reduction of the solute carbon and the nitrogen contents in the ferrite matrix caused by the precipitation of BN, as well by the increase in the volume fraction of the cementites.

## Abstract 2

The effect of nano-sized precipitates on the mechanical properties of boron-added low-carbon steels was studied by neutron scattering techniques such as powder diffraction, small-angle scattering and particle tracking autography.

## 1. Introduction

It is important to control the precipitates and solute elements in low-carbon steels during heat treatment to obtain materials with high formability and high strength for use in automotive parts. Many studies have been carried out (Shin *et al.*, 2003 ▶; Carabajar *et al.*, 2000 ▶; Hinotani *et al.*, 1994 ▶; Rege *et al.*, 2000 ▶) to investigate the effects of precipitates and solute elements such as carbon, nitrogen, sulfur and phosphorus on mechanical properties.

The above solute elements form precipitates of different types, such as TiC, TiN, TiS and Ti_4_C_2_S_2_ (Shin *et al.*, 2003 ▶; Carabajar *et al.*, 2000 ▶; Mendoza *et al.*, 2000 ▶). These precipitates eliminate solute elements in the ferrite matrix. A reduction of the interstitial content reduces the strength of the steels and increases their formability. One way to increase the strength of low-carbon steels is to add solid solution strengthening elements.

The addition of boron to low-carbon steels increases their hardenability as a result of its segregation at the austenite grain boundaries. This reduces the ferrite nucleation rate during an austenite–ferrite phase transformation, which suppresses the formation of polygonal ferrite (Funakawa *et al.*, 2001 ▶; Tsuji *et al.*, 1997 ▶). However, several studies have reported that the *R* value of low-carbon steels decreases with boron addition (Haga *et al.*, 1998 ▶; Hosoya *et al.*, 1993 ▶). The mechanism by which boron increases the hardenability is not clear. While the effect of boron on the tensile strength is known through its influence on the hardenability, its influence on elongation is still a controversial issue (Haji & Wakita, 2000 ▶).

In this study, the effects of nano-sized precipitates and boron addition on the mechanical properties of low-carbon steels were investigated quantitatively in order to understand the development of precipitation behaviour with hot-rolling temperature. Small-angle neutron scattering (SANS), neutron powder diffraction (ND) and particle tracking autoradiography (PTA) techniques were used to investigate quantitatively the precipitation behaviours in boron-free (BF) and boron-added (BA) low-carbon steels. The size distribution and volume fraction of nano-sized precipitates such as MnS and BN, which are precipitated during the hot-rolling process in BA steel, were evaluated quantitatively using SANS techniques. The weight fraction of the cementite precipitates was estimated by the Rietveld analysis method (Rietveld, 1969 ▶; Rodriguez-Carvajal, 1998 ▶). The effects of boron addition and of boron nitride precipitates on the mechanical properties of low-carbon steels will be discussed.

## 2. Experimental

BA and BF low-carbon steels produced by the POSCO Steel Company, Korea, were used in the present work. The chemical compositions of the steels are given in Table 1 ▶. The ingots were melted in a vacuum and hot-rolled to form small slabs with a thickness of 30 mm after heating to 1473 K. The slabs were reheated to 1423 K for 120 min. After heat treatment, a seven-pass hot strip-rolling experiment was performed to obtain a 2.6 mm thick strip. The final rolling speed was set at 150 m min^−1^, which is equivalent to a rolling speed of 2.5 m s^−1^. In order to investigate the effect of the hot-rolling temperature, hot-rolling was performed over a final-pass temperature (FET) range of 1133–1193 K. After hot-rolling, the hot-rolled sheets were cooled to 923 K, corresponding to a coiling temperature.

Microstructure observation was performed with optical microscopy (Nikon 2400) and transmission electron microscopy (TEM; Philips CM1200). A replica method was used in TEM analysis. To measure mechanical properties, a tension test (Zwick, 10 ton) was carried out at a cross-head speed of 5 mm min^−1^ for a plate shape 50 mm long and 25 mm wide.

ND and SANS techniques were used to determine the total weight fraction of the cementite precipitates and the volume fraction and distribution of the nano-sized precipitates of less than about 600 Å in diameter. ND patterns were obtained using a high-resolution neutron powder diffractometer (Δ*d*/*d* ≃ 2 × 10^−3^) equipped with 32 detectors at the HANARO reactor at the Korea Atomic Energy Research Institute, Korea. The neutron wavelength was 1.84 Å. Data were collected at intervals of 0.05° between 20 and 155° in 2θ. The PTA method was also used to clarify boron distribution with hot-rolling temperature in BA steels. (Shin *et al.*, 2008 ▶)

**Figure 1:**
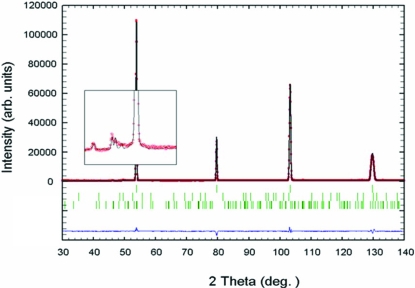
Rietveld refinement results for the BF low-carbon steel with a rolling temperature of 1148 K. Inset shows the refinement results of minor cementite phases.

In order to evaluate the total weight fraction of cementite in the BF and BA steels, ND was combined with Rietveld refinement. As an example of Rietveld refinement, the ND pattern of BF steel with a rolling temperature of 1148 K is shown in Fig. 1 ▶. The observed intensities are depicted by circles and the upper solid line represents the calculated fits. The three rows of small bars represent the Bragg reflection positions of the bcc phase (ferrite and bainite), the bainite–cementite phase in space group *P*6_3_22 and the cementite phase in space group *Pbnm*. The lower solid line represents the differences between the observed and calculated intensities. The inset represents the Rietveld refinement result of some cementite peaks. Even though the cementite peaks are very weak, the calculated data seem to be in good agreement with the measured data. The refined lattice parameters of the cementite phase (*Pbnm*) are *a* = 4.65, *b* = 5.13 and *c* = 8.10 Å. As this *c* value is larger than that of *c* = 6.74 Å given in the literature (Cullity, 1978 ▶), it appears that a metastable phase forms during the hot-rolling process.

SANS experiments with a magnetic field of 1.0 T were performed at the HANARO reactor (Seong *et al.*, 2002 ▶). The wavelength used was λ = 5.08 Å, the sample-to-detector distance was 3.0 m and the wavelength spread (Δλ/λ) was about 12%. Defining the scattering vector 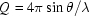, where 2θ is the scattering angle, the intensity was obtained at *Q* values ranging between 0.01 and 0.12 Å^−1^, corresponding to a precipitate size of 50–600 Å in real space. Two-dimensional scattering patterns collected by two-dimensional area detectors with an active area of 64.5 × 64.5 cm were averaged to produce a one-dimensional intensity profile. The scattering intensity data were reduced to an absolute scale using standard porous silica in a 1 mm path-length quartz cell (Seong *et al.*, 2002 ▶) and by means of ILL standard programs (Ghosh *et al.*, 2006 ▶).

In the case of steel samples, the total SANS cross section dΣ(*Q*)/dΩ (where Ω stands for the solid angle) can be written as 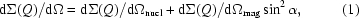
where 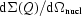 and 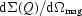 are the nuclear and magnetic SANS cross section, respectively, and α is the azimuthal angle on the detector plane. When a saturating horizontal magnetic field of 1.0 T perpendicular to the incident neutron beam is applied, we can distinguish 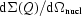 and 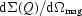 from dΣ(Q)/dΩ. Nuclear scattering only occurs in the horizontal plane, though nuclear and magnetic scattering occur in the vertical plane. In order to understand the effect of nano-sized precipitation behaviour in low-carbon steels, only nuclear scattering can be considered.

**Figure 2:**
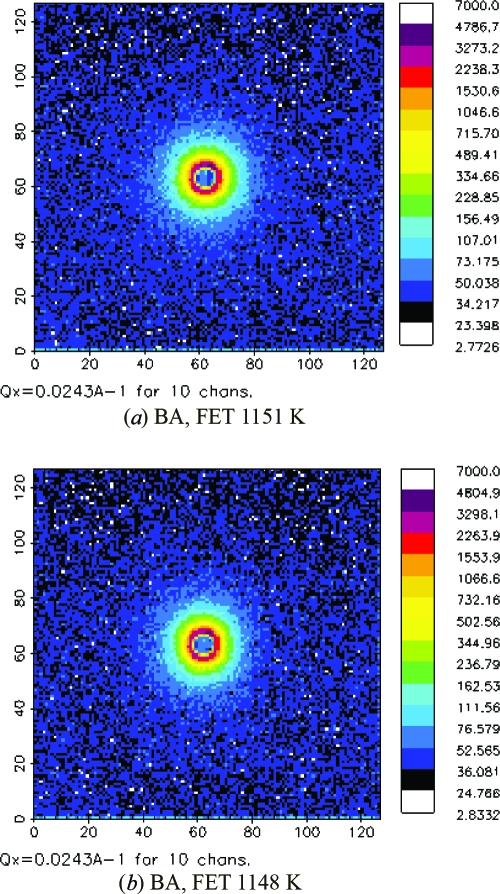
Two-dimensional SANS spectra obtained with a magnetic field of 1.2 T for (*a*) BA, FET 1151 K and (*b*) BF, FET 1148 K samples.

**Figure 3:**
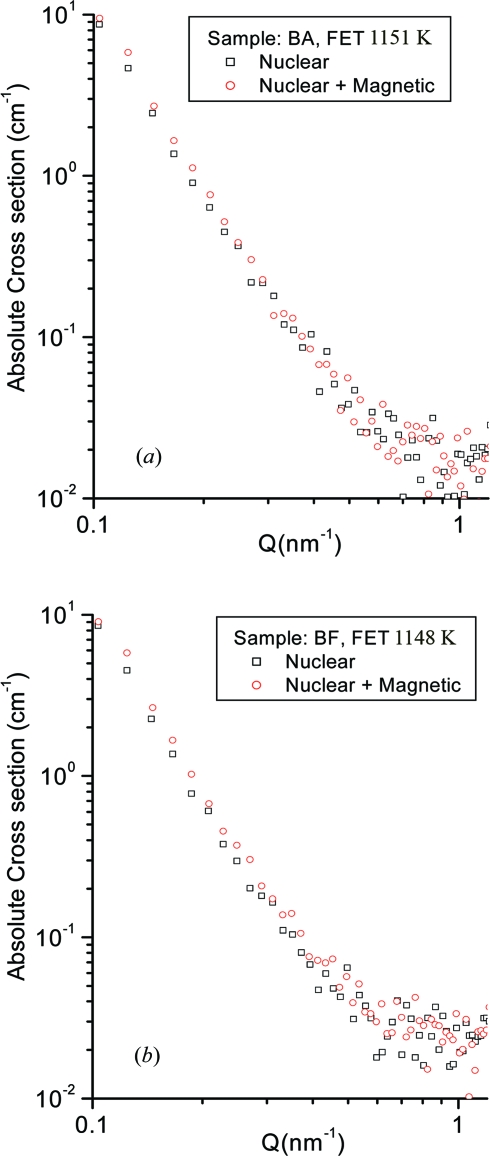
Nuclear scattering cross sections (open squares) and nuclear plus magnetic scattering cross sections (open circles) for (*a*) BA, FET 1151 K and (*b*) BF, FET 1148 K samples.

Fig. 2 ▶ shows an example of the two-dimensional SANS spectra obtained with a magnetic field of 1.0 T for the BA and BF steels. It is seen that the patterns in both samples are isotropic. In order to investigate the effect of magnetic scattering on the SANS patterns, the nuclear scattering contributions (open squares) extracted from the two-dimensional SANS patterns are compared with nuclear plus magnetic scattering contributions (open circles), as shown in Fig. 3 ▶. In both the BA and the BF steels, the magnetic scattering is not strong over all the *Q* ranges, and the contributions from the magnetic scattering can be negligible in the range *Q* > 0.03 Å^−1^, corresponding to a precipitate size of < 200 Å.

## 3. Results and discussion

**Figure 4:**
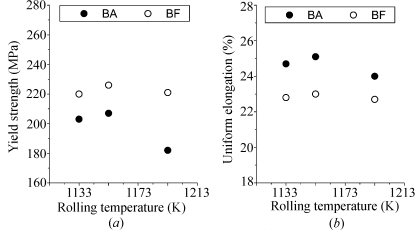
Effects of the rolling temperature on the mechanical properties of the low-carbon steel: (*a*) yield strength and (*b*) uniform elongation.

**Figure 5:**
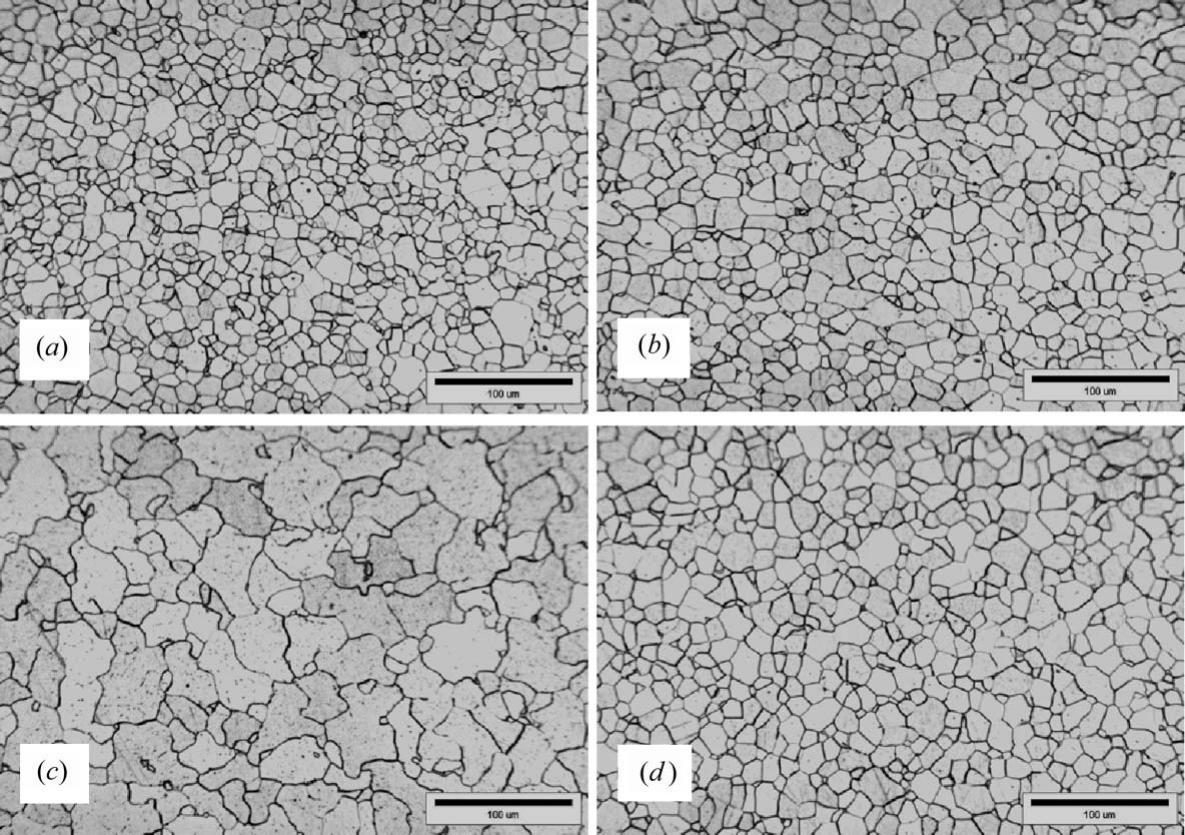
Optical microstructures of hot-rolled sheets for (*a*) and (*c*) BA, and (*b*) and (*d*) BF low-carbon steels. The rolling temperature in views (*a*) and (*b*) was 1148 K, while in views (*c*) and (*d*) it was 1193 K.

The effect of the hot-rolling temperature on the yield strength and uniform elongation of BF and BA steels is shown in Figs. 4 ▶(*a*) and 4 ▶(*b*), respectively. Boron addition decreases the yield strength and increases the uniform elongation. The yield strength and uniform elongation of the BF steels maintain nearly constant values with the hot-rolling temperature, whereas the yield strength of BA steels decreases drastically at a higher rolling temperature. Such dependency of the mechanical properties on the hot-rolling temperature for BA steels is suggested to be due to the different microstructural evolution and precipitation behaviour of these steels. The ferrite grains in the BA steels become coarse and irregular at a higher rolling temperature, as shown in Fig. 5 ▶, whereas the ferrite grains in the BF steels did not change significantly with the rolling temperature.

**Figure 6:**
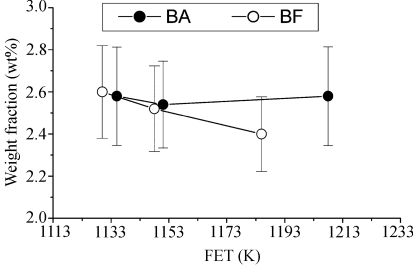
Total weight fraction of the cementite obtained from the Rietveld analysis *versus* rolling temperature (FET) for low-carbon steels.

The total weight fraction of the cementites obtained from the Rietveld analysis with rolling temperature is shown in Fig. 6 ▶. In the BA steels, the weight fraction of the cementite did not change significantly. On the other hand, in the BF steels, the weight fraction of the cementite gradually increases with decreasing rolling temperature.

**Figure 7:**
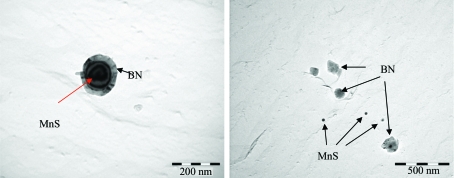
TEM bright-field images of the precipitates for the BA low-carbon steels.

Two TEM bright-field images of the precipitates for the BA steels are shown in Fig. 7 ▶. Most of the precipitates in the ferrite matrix were identified by EDS analysis as BN precipitates which have a nucleus of MnS or CuS. The nucleus size is mainly in the range 100–500 Å, although some precipitates contain a large nucleus of up to 5000 Å. In addition, BN precipitates have grown in particular on the surface of the MnS or CuS nuclei. Thus, in the BA steels, the shapes of the precipitates are either a core–shell structured sphere or an ellipsoidal shape, such as MnS and CuS precipitates surrounded by BN layers. The shapes of precipitates like MnS and CuS are either spherical or ellipsoidal in the BF steels.

**Figure 8:**
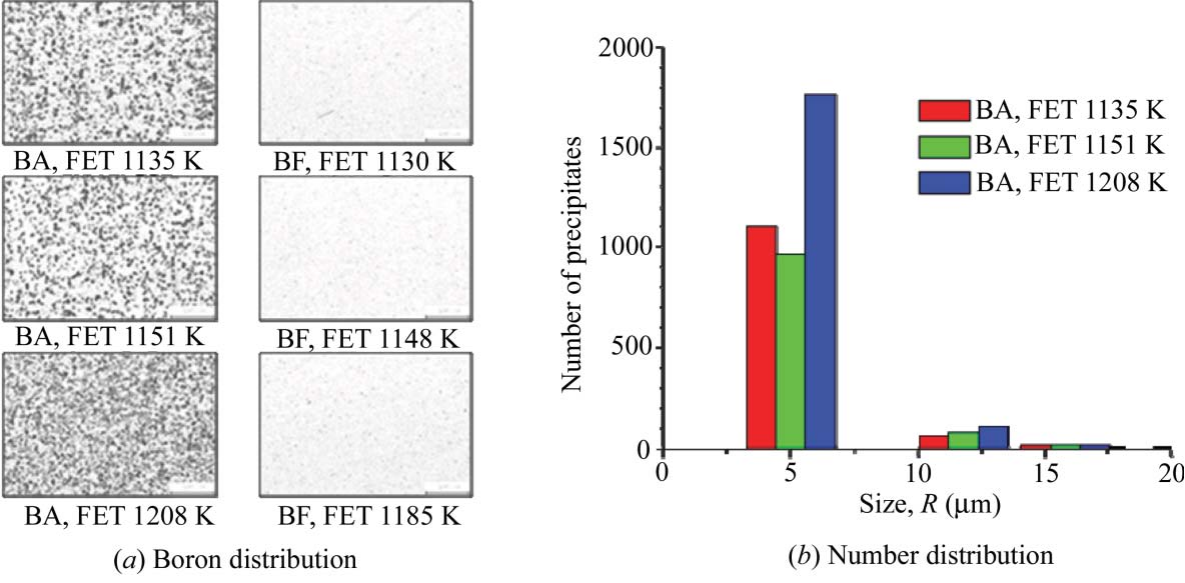
(*a*) Boron distribution and (*b*) number of boron precipitates such as BN, MnS surrounded by BN, Fe_3_(C,B) *etc.* *versus* rolling temperatures (FET) for the BA and BF steels measured by the PTA method.

Fig. 8 ▶ shows (*a*) the boron distribution and (*b*) the number of boron precipitates, assuming that the precipitates are spherical, in the BA and BF steels measured by the PTA method. It is clearly shown that, in the BA steels, boron precipitates such as BN, Fe_3_(C,B) and MnS surrounded by BN layers are detected and the number of boron precipitates smaller than 5 µm increases drastically at higher hot-rolling temperatures, whereas in BF steels no boron precipitate was detected.

**Figure 9:**
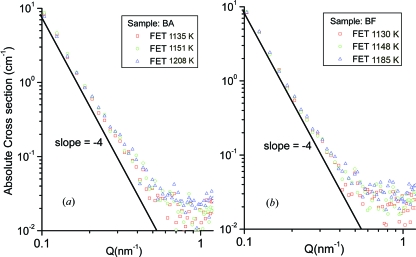
Measured nuclear SANS cross section for low-carbon steels: (*a*) BA steels and (*b*) BF steels.

The one-dimensional nuclear SANS cross sections extracted from the two-dimensional SANS patterns for (*a*) the BA and (*b*) the BF steels for different hot-rolling temperatures are shown in Fig. 9 ▶. The intensities of the SANS spectra show a small difference over all the measured *Q* ranges in the BA steels according to the hot-rolling temperature, whereas the intensities do not show a significant difference in the BF steels. However, in both the BA and the BF steels, the intensities follow the law ∼*A*_p_ *Q* ^−4^ for *Q* < 0.02 Å^−1^, known as Porod (1982 ▶) scattering. *A*_p_ is proportional to the total area of the interface between the precipitates and the matrix. The intensities in the *Q* range 0.02–0.06 Å^−1^ for all the samples show a clear difference due to the polydispersed precipitates. The intensities for *Q* > 0.11 Å^−1^ become close to the background level.

The scattering length densities of possible fine scatterers such as MnS, BN, Fe_3_C and Fe_3_B precipitates, which can contribute to SANS intensities for *Q* > 0.02 Å^−1^, are η_MnS_ = −0.25 × 10^10^ cm^−2^, η_BN_ = 12.0 × 10^10^ cm^−2^, η_Fe3C_ = 9.0 × 10^10^ cm^−2^ and η_Fe3B_ = 8.7 × 10^10^ cm^−2^, respectively. The squared scattering contrast between the pure Fe matrix and MnS and BN precipitates is higher by a factor of between 10 and 100 than that for Fe_3_C and Fe_3_B precipitates. Thus, the SANS intensities were mainly attributed to MnS and/or MnS surrounded by BN layers, as shown in Fig. 7 ▶.

In the case of the BF steels, the intensities of the nuclear SANS spectra are affected by MnS precipitates of less than 200 Å. The macroscopic differential scattering cross section due to the above spherical precipitates is, therefore, given by (Kohbrecher, 1999 ▶; Keiderling *et al.*, 2000 ▶; Shin *et al.*, 2003 ▶)
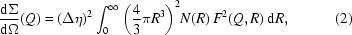
where (Δη)^2^ is the scattering contrast (η is the scattering length density), depending on the chemical composition of both the precipitates and the matrix, and *R* is the radius of the spherical precipitates. *F* is the form factor of the spherical precipitates, and *N*(*R*)d*R* is the number of precipitates with typical size between *R* and *R* + d*R* per unit volume. However, in the case of the BA steels, the intensities of the nuclear SANS spectra are affected by the MnS precipitate, which is surrounded by BN layers as shown in Fig. 7 ▶, *i.e.* spherical core–shell structured precipitates, and by small MnS or/and CuS precipitates of less than 600 Å. The macroscopic differential scattering cross section due to the above spherical core–shell structured precipitates is given by (Kohbrecher, 1999 ▶; Keiderling *et al.*, 2000 ▶)
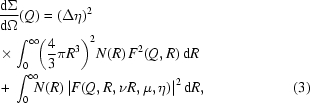
where *R* is the radius of the shell, ν*R* is the core radius, η(1−μ) is the neutron scattering length density of the core and η is the neutron scattering length density of the shell. *F* is the form factor of the core–shell structure, given by 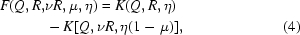
where 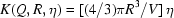 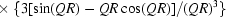.

Core–shell structured precipitates of less than 200 Å were dispersed in the BA steels. In the BF steels, MnS precipitates with a wider size range were dispersed. The scattering length densities of the MnS and BN precipitates in the iron matrix are (*Δη*)_MnS_ = 8.3 × 10^10^ cm^−2^ and (*Δη*)_BN_ = 4.4 × 10^10^ cm^−2^, respectively (Mittra *et al.*, 2004 ▶).

A model fitting of the real-space size distribution to the SANS spectra was performed using a nonlinear least-squares method (Kohbrecher, 1999 ▶). A simple real-space model consists of a spherical (for the BF steels) or a core–shell structured spherical (for the BA steels) distribution of the precipitates per curve. These log-normal distributions were fitted to the *Q* range between 0.01 and 0.12 Å^−1^ in equation (2) for the BF steels and in equation (3) for the BA steels. The Porod background, *I*(*Q*) ≃ *A*_p_ *Q* ^−4^ + bkg, was used for model fitting. The Porod constant *A*_p_ is closely related to the scattering contrast factor and total interface area between the precipitates and the matrix, and bkg is proportional to the measured incoherent scattering due mostly to nuclei concentrations and the contributions of small defects such as vacancies.

**Figure 10:**
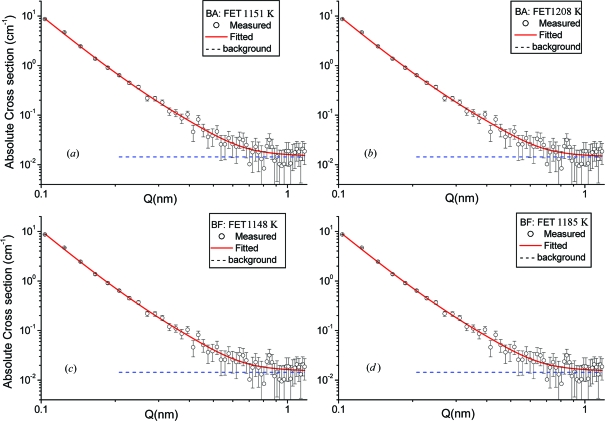
Measured nuclear SANS data and fitted results. (*a*) BA, FET 1151 K, (*b*) BA, FET 1208 K, (*c*) BF, FET 1148 K and (*d*) BF, FET 1185 K.

Some examples of the measured nuclear SANS spectra and the fitted spectra for the BA and BF steels are shown in Fig. 10 ▶. Open circles correspond to the observed intensities and the solid line represents the fitted ones. Dashed lines are backgrounds. Even though the spectra are very weak and have poor statistics above 0.05 Å^−1^, the fitted spectra are in good agreement with the measured ones. As model fitting results, the Porod constants *A*_p_ and the bkg values were obtained as almost same values, 1.013 × 10^25^ cm^−5^ and 0.0146 cm^−1^, respectively, for all samples. This means that all samples have similar large precipitates with almost the same compositions of the precipitates and interface area between the matrix and precipitates.

**Figure 11:**
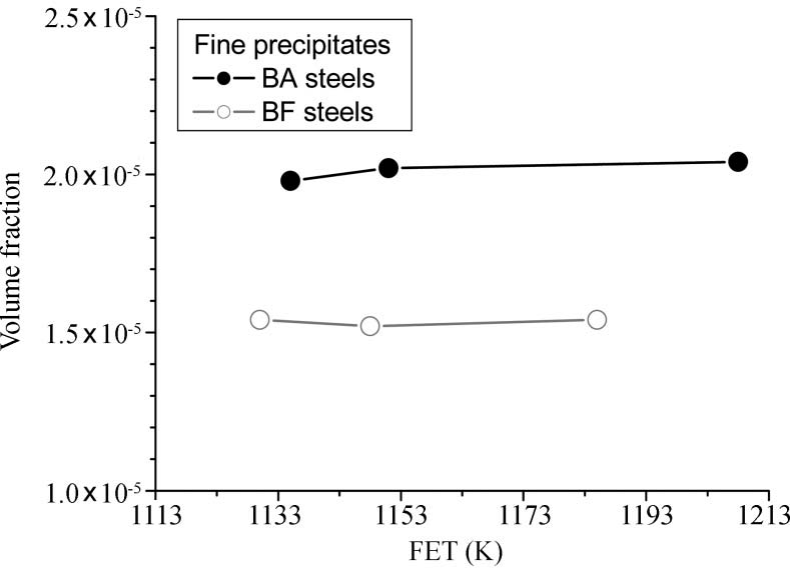
Volume fraction of the precipitates *versus* rolling temperature (FET) for the BA and BF steels.

Figs. 11 ▶ and 12 ▶ show, respectively, the volume fraction and average radius of the precipitates calculated by a direct model fitting of the SANS spectra as a function of the hot-rolling temperature for the BA and BF steels. The results reveal that the volume fraction of the precipitates in the BA steels is larger than that in the BF steels. Fine spherical core–shell structured precipitates, with an average radius of ∼50 Å, exist in the BA samples, whereas fine spherical precipitates, with an average size of ∼48 Å, exist in the BF steels. When boron is added to low-carbon steels, the precipitates are coarsened by the growing BN layers on the MnS and CuS precipitates. However, the average size of fine precipitates less than 200 Å in radius in both the BA and the BF steels exhibited no significant changes with decreasing rolling temperature.

It is also found that, when boron is added, the volume fraction of the fine precipitates in the low-carbon steels increases. There are two reasons for this: the BN layers grow rapidly on the MnS or CuS precipitates, as shown in Fig. 11 ▶, and the weight fraction of boron–cementite phases like Fe_3_(C,B) larger than 200 Å in radius increases with increasing rolling temperature because of a decrease in the weight fraction of the cementite phases, as shown in Fig. 6 ▶. The number of boron precipitates such as BN and MnS surrounded by BN, Fe_3_(C,B) and so on, also increases at higher rolling temperature, as shown in Fig. 8 ▶. However, in the BF steels, MnS and CuS precipitates are observed and the volume fraction of the precipitates less than 200 Å in radius does not change significantly. This suggests that the precipitation of the boron precipitates is activated at a higher rolling temperature. Thus, the excess boron reduces and the excess carbon/nitrogen increases a little in the matrix. In addition, the ferrite grain size of the BA steels was larger than that of the BF steels and the coarsening behaviour of ferrite was noticeable at a higher rolling temperature, as shown in Fig. 5 ▶.

It is clear that boron addition to low-carbon steels increases the uniform elongation and decreases the yield strength, as shown in Fig. 4 ▶, and that it increases the volume fraction of both the spherical core–shell structured precipitates, as shown in Fig. 11 ▶, and the cementites, as shown in Fig. 6 ▶. In the BA steels, it seems that the coarsening of the BN precipitates having fine nuclei of MnS or CuS and the increased volume fraction of the cementites reduced the solute nitrogen and carbon contents. As a result, the BA steels exhibited lower strength and higher elongation values than the BF steels.

## 4. Conclusions

The effects of nano-sized precipitates and boron addition on the mechanical properties of low-carbon steels have been investigated quantitatively using SANS, ND and PTA techniques. Fine precipitates, with an average radius of ∼48 and ∼50 Å, exist in the BF and BA steels, respectively. The fine precipitates in the BA steels have a spherical core–shell structured shape, which consists of MnS precipitates surrounded by BN layers, whereas the precipitates in the BF steels are spherical structures.

In BA steels the number of boron precipitates such as BN, Fe_3_(C,B) and MnS surrounded by BN drastically increases at higher hot-rolling temperature. It is also found that the volume fraction of the precipitates in the BA steels is higher than that in the BF steels because the BN layers grow rapidly on the MnS or/and CuS precipitates.

Boron addition to low-carbon steels can play a role in decreasing the strength and improving the elongation; this result is due to the reduction of the solute nitrogen and carbon contents, *i.e.* scavenge effects in the ferrite matrix, caused by a precipitation of the BN precipitates, as well as to the increasing volume fraction of the cementites.

## Figures and Tables

**Figure 12:**
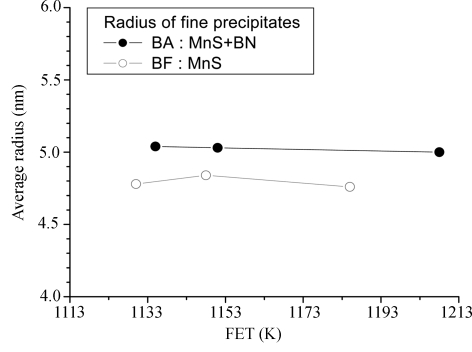
Average radius of the precipitates *versus* rolling temperature (FET) for the BA and BF steels.

**Table 1:** Chemical compositions of the low-carbon steels

	Chemical composition (wt%)						
Steel	C	Mn	P	S (p.p.m.)	Sol-Al	B (p.p.m.)	N (p.p.m.)
BF	0.020	0.20	0.012	40	0.025	–	20
BA	0.020	0.20	0.011	40	0.025	20	20
